# Influential Factors, Treatment and Prognosis of Autoimmune Encephalitis Patients With Poor Response to Short-Term First-Line Treatment

**DOI:** 10.3389/fneur.2022.861988

**Published:** 2022-04-14

**Authors:** Fei Liu, Bingbing Zhang, Teng Huang, Baojie Wang, Chunjuan Wang, Maolin Hao, Shougang Guo

**Affiliations:** ^1^Department of Neurology, Shandong Second Provincial General Hospital, Jinan, China; ^2^Department of Neurology, Shandong Provincial Hospital, Cheeloo College of Medicine, Shandong University, Jinan, China; ^3^Department of Resident Trainint, Qilu Hospital of Shandong University, Jinan, China; ^4^Department of Neurology, Shandong Provincial Hospital, Shandong First Medical University, Jinan, China

**Keywords:** autoimmune encephalitis, characteristics, treatment, response, prognosis

## Abstract

**Objective:**

This study was performed to assess the potential factors for poor short-term first-line treatment response, the appropriate further treatment options, and the prognosis in patients with autoimmune encephalitis (AE).

**Methods:**

This retrospective study consisted of 135 patients with AE. According to their short-term first-line treatment response, patients were divided into the response group and the non-response group. The demographics, clinical characteristics, main accessory examinations, immunotherapy, and outcomes of patients were compared between the two groups. Univariate and multivariate logistic regression models were used to analyze whether non-responders have poor long-term outcomes. Further treatment and prognosis of non-responders were also analyzed.

**Results:**

Of the 128 patients who were treated with first-line immunotherapy, 59 (46.1%) were non-responders. Patients in the non-response group had more symptoms and exhibited a higher proportion of mental behavior disorder, central hypoventilation, and autonomic nervous dysfunction. The modified Rankin scale (mRS) scores and neutrophil-to-lymphocyte ratio (NLR) levels were significantly higher and albumin, high-density lipoprotein cholesterol (HDL-C), apolipoprotein A (apoA) levels were significantly lower in the non-response group (*p* < 0.05, all). Multivariate logistic regression analysis showed that the number of clinical symptoms, mental behavior disorder, central hypoventilation, maximum mRS score, and albumin level was independently associated with non-response to short-term first-line treatment. Non-responders had poor long-term outcomes compared with the responders at all times of followed-up (*p* < 0.05, all). In multivariable analysis, initial first-line treatment response was independently associated with the long-term prognosis, both at 12-month [odds ratio (OR), 4.74, 95% CI, 1.44–15.59, and *p*=0.010] and 24-month follow-ups (OR, 8.81, 95% CI, 1.65–47.16; and *p* = 0.011). Among the non-responders, a higher improvement of mRS scores was observed in those who received second-line treatment than those who had no further treatment or repetition of first-line immunotherapy in the follow-up. However, the rate of a good outcome and median mRS scores were not significantly different among the three groups.

**Conclusion:**

Disease severity, clinical features, anti-N-methyl-D-aspartate receptor subtypes, antibody titers, NLR, albumin, HDL-C, and apoA levels were all associated with non-response to short-term first-line treatment. The short-term first-line treatment response is a valuable predictor of long-term outcomes in patients with AE. Second-line immunotherapy may be a more aggressive treatment option for patients who failed short-term first-line immunotherapy.

## Introduction

Autoimmune encephalitis (AE) is a potentially life-threatening neurologic disease associated with brain inflammation and antibodies against specific brain antigens (1). The clinical manifestations of patients with AE are broad and include a series of subacute, severe, and complex neuropsychiatric symptoms, such as cognitive impairment, mental behavioral disorder, epileptic seizures, autonomic nervous dysfunction, consciousness change, and central hypoventilation (2–4). Recent research found that mortality of patients with AE ranged between 11 and 18%, up to 56% of patients were reported to be severely disabled at discharge, and about 20% of patients still had a poor prognosis at long-term follow-up (4–7). Fortunately, the disease is treatable, and within recent years, several therapies have been discovered. Retrospective observations indicate that early aggressive treatment is associated with better functional outcomes and fewer relapses (2, 4). Approved therapies for AE include first-line immunotherapy (corticosteroids, intravenous immunoglobulin, plasma exchange, and immunoadsorption) and second-line immunotherapy (rituximab, cyclophosphamide, azathioprine, tocilizumab, and bortezomib) (8, 9). However, in individual patients, the course of the disease and the response to treatment are different and remain largely unpredictable at the onset of the disease. It is important for clinicians to be able to predict the individual patient's response to therapy early, to make a quick optimal therapeutic decision.

Previous studies found that patients with central hypoventilation, autonomic dysregulation, epilepsy, involuntary movement, and disturbance of consciousness appear to indicate severity and poor prognosis (5, 10). Early aggressive treatment is associated with good outcomes (2, 11). Second-line immunotherapy is usually effective when first-line treatments failed (4, 12, 13). However, there are limited data concerning the short-term treatment response of first-line immunotherapy and its impact on the long-term prognosis of patients with AE. Existing studies also differ in the observation time of first-line treatment effect evaluation, with many studies taking 4 weeks or more (4, 14, 15). The assessment time is too long for patients admitted to our facility. Dalmau et al. (12) recommended that second-line treatment should be added if first-line treatment failed after 10 days. Recent studies indicate that patients with the early addition of second-line treatment often have a good prognosis (16, 17). It is of great significance to identify the factors affecting short-term first-line treatment response early and adjust treatment timely.

Therefore, this study focuses on the clinical characteristics and prognosis of the patients with AE who failed short-term (10–14 days) first-line immunotherapy. Our study aimed to assess the potential factors for nonresponse to short-term treatment, the appropriate further treatment options, and the long-term prognosis impact in patients with AE. This study could help neurologists predict the treatment response and prognosis early in patients with AE, and adjust appropriate treatment timely, which may significantly improve the outcomes of these patients.

## Methods

### Ethical Approval

This retrospective study was approved by the ethics committee of the Shandong Provincial Hospital Affiliated to Shandong University and was performed in accordance with the ethical standards laid down in the 1964 Declaration of Helsinki and its later amendments. Patient identity remained anonymous, and the requirement for informed consent was waived due to the observational nature of the study.

### Study Design and Participants

In this retrospective study, patients who were diagnosed as possible AE and hospitalized in the Shandong Provincial Hospital Affiliated to Shandong University from September 2014 to July 2021 were enrolled. All patients were screened at least once for tumors. The patients' routine examinations included brain MRI, CT scan of the thorax/abdomen/pelvis, B ultrasound of the abdomen and pelvic region, electroencephalogram (EEG), cerebrospinal fluid (CSF), and blood examinations. All patients' autoantibodies testing in both serum and CSF were performed through indirect immunofluorescence testing by third-party medical testing agencies. Only the patients who tested positive for autoantibodies against neuronal surface or synaptic proteins were included in the study. The antibody panel included anti-N-methyl-D-aspartate receptor (NMDAR), anti-gamma-aminobutyric acid B receptor (GABABR), anti-leucine-rich glioma inactivated protein 1 (LGI1), anti-contactin-associated protein-like 2 (CASPR2), and myelin oligodendrocyte glycoprotein (MOG). All patients fulfilled the diagnostic criteria for AE (18). We excluded patients if they had received immunotherapy before this study. Patients with incomplete clinical data were also excluded.

### Data Collection

Clinical information, namely, patients' demographics information (sex, age, height, and weight), medical history, prodromal symptoms at presentation (fever, headache, respiratory symptoms, emesis, and diarrhea), clinical manifestations (mental behavior disorder, cognitive impairment, epileptic seizure, consciousness change, autonomic nervous dysfunction, and central hypoventilation), and auxiliary examination results were obtained from the patients' medical records by two investigators. According to levels of antibody titers, samples were classified as weakly positive (1:10), positive (1:32), and strongly positive (titer of 1:100 or above).

Venous blood samples were drawn by venipuncture in the morning after an overnight fast at least 8 h after admission. Blood examinations mainly include the following items: C-reactive protein, neutrophil-to-lymphocyte ratio (NLR), albumin, total bilirubin, homocysteine, total cholesterol, triglycerides, low-density lipoprotein cholesterol, high-density lipoprotein cholesterol (HDL-C), apolipoprotein A (apoA), apolipoprotein B (apoB), and apoA/apoB levels.

### Treatment and Prognosis Evaluation

All patients received immunotherapy and symptomatic supportive treatment. First-line immunotherapy was defined as the use of intravenous glucocorticoid therapy and intravenous gamma immunoglobulin (IVIG), alone or combined. No plasma exchange and immunoadsorption were performed due to the limitations of our hospital. Second-line immunotherapy included rituximab, cyclophosphamide, and bortezomib, alone or combined. We assessed the patients' neurological status with the modified Rankin scale (mRS) (19). We recorded short-term first-line immunotherapy as non-response or failed if no sustained improvement occurred within 10–14 days after initiation of one round of first-line immunotherapy or tumor removal and if the mRS score remained at 4 or higher. According to the short-term first-line treatment response, patients were divided into the response group and the non-response group. All patients received outcome evaluations at discharge and every time of follow-up. We obtained follow-up information at regular intervals after discharge (months 2, 4, 6, 12, 18, and 24). In this study, an mRS score of 0–2 is considered “good outcome” and 3–6 points as “poor outcome.” Relapse was defined as the appearance of new symptoms or the worsening of pre-existing symptoms after improvement or stabilization of the disorder for at least 2 months, not explained by other causes. Improvement of the mRS score was calculated by subtracting the mRS score at follow-up from the mRS score at the end of short-term first-line therapy.

### Statistical Analysis

All statistical analyses were performed using SPSS 19.0 software (SPSS Inc., Chicago, IL, USA). *p* < 0.05 were considered statistically significant.

Data with normal distributions are expressed as the mean ± SD, whereas data with non-normal distributions are expressed as the median (interquartile range, IQR). Categorical variables were expressed as counts (percentages). Student's *t*-test or one-way ANOVA was used for intergroup comparisons of data with a normal distribution and homogeneous variance, otherwise, the Mann–Whitney *U* test or Kruskal–Wallis test was used. The chi-square test or Fisher's exact test was used for intergroup comparisons of categorical variables. And the candidate variables with a univariate relationship (*p* < 0.06) with non-response to short-term first-line treatment were included in a multivariate logistic regression to determine the independent predictors of non-response. A univariate and multivariate logistic regression analysis was conducted to evaluate whether short-term first-line treatment response can predict the long-term outcomes.

## Results

### Clinical Characteristics

**Figure 1:**
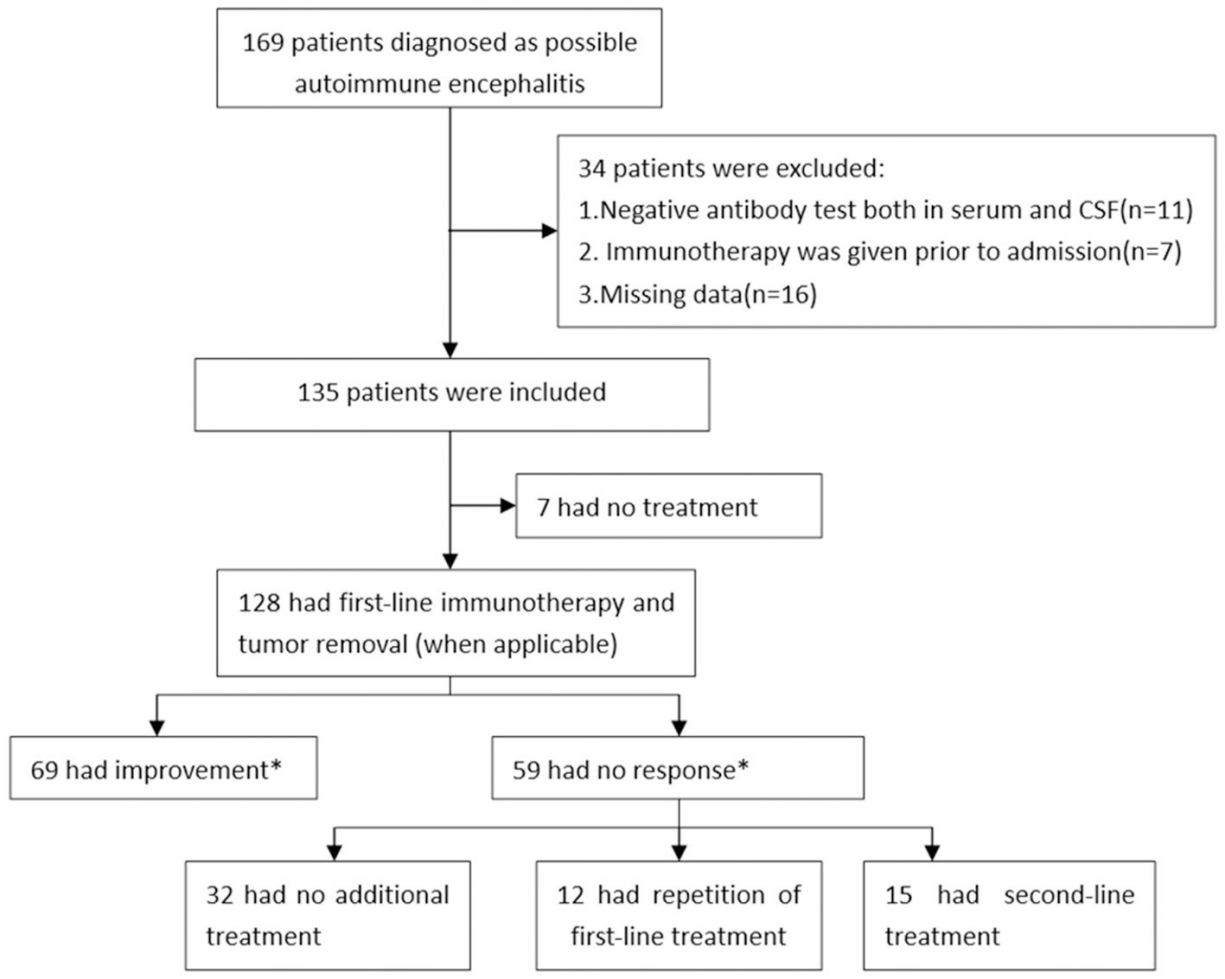
Study profile in autoimmune encephalitis patients. *The occurrence of improvement was assessed at 10–14 days from initiation of first-line treatment.

Overall, we enrolled 135 patients with AE in this study and 128 patients had first-line immunotherapy. Based on their response to the short-term first-line immunotherapy, 69 (53.9%) patients were in the response group, and 59 (46.1%) were in the non-response group (Figure 1). Table 1 summarized the detailed clinical characteristics of these patients. The median age of all patients at study entry was 48 years (IQR: 30–58 years), and 62 patients (45.9%) were women. The median BMI of patients was 23.33 Kg/m^2^ (IQR: 21.48–25.06 Kg/m^2^).

**Table 1:** Demographic and clinical characteristics of patients with autoimmune encephalitis.

**Variable**	**All (*n* = 135)**	**Responses group (*n* = 69)**	**Non-response group (*n* = 59)**	***P*** **-value**
Age, years	48 (30, 58)	48 (31–58)	49 (32–62)	0.761
Sex, female, *n* (%)	62 (45.9)	31 (44.9)	28 (47.5)	0.775
BMI, Kg/m^2^	23.33 (21.48, 25.06)	23.56 (21.62–24.82)	22.95 (20.96–25.47)	0.867
Median time from symptom onset until treatment, days	26(13–63)	30 (14–150)	22 (10–33)	0.053
Prodromal symptoms at presentation, *n* (%)	48 (35.6)	23 (33.3)	24 (40.7)	0.390
Number of clinical symptoms	3 (2–4)	3 (2–4)	4 (3–5)	<0.001
Clinical symptoms, *n* (%)				
Mental behavior disorder	77 (57.0)	31 (44.9)	44 (74.6)	0.001
Epileptic seizure	103 (76.3)	55 (79.7)	42 (71.2)	0.262
Disturbance of consciousness	95 (70.4)	43 (62.3)	46 (78.0)	0.055
Cognitive impairment	122 (90.4)	63 (91.3)	54 (91.5)	0.965
Central hypoventilation	15 (11.1)	1 (1.4)	14 (23.7)	<0.001
Autonomic nervous dysfunction	48 (35.6)	17 (24.6)	31 (52.5)	0.001
MRS at study entry, scores	4 (3–4)	4 (4–4)	4 (4–5)	0.032
Maximum mRS, scores	4 (4–5)	4 (4–4)	5 (4–5)	<0.001
Antibodies, *n*, (%)				0.023
NMDAR	57 (42.2)	24 (34.8)	31 (52.5)	0.043
LGI-1	43 (31.9)	29 (42.0)	11 (18.6)	0.004
GABABR	20 (14.8)	7 (10.1)	13 (22.0)	0.065
CASPR2	8 (5.9)	4 (5.8)	3 (5.1)	1.000
MOG	5 (3.7)	4 (5.8)	1 (1.7)	0.373
Antibody titers, *n*, (%)				0.015
Weakly positive	47 (34.8)	25 (36.2)	16 (27.1)	0.271
Positive	63 (46.7)	37 (53.6)	25 (42.4)	0.204
Strongly positive	25 (18.5)	7 (10.1)	18 (30.5)	0.004
Abnormal EEG, *n* (%)	94 (69.6)	50 (72.5)	39 (66.1)	0.436
Tumor comorbidity, *n* (%)	22 (16.3)	9 (13.0)	13 (22.0)	0.179
CRP, mg/L	2.01 (0.63–7.71)	1.32 (0.61–6.70)	2.91 (0.65–9.74)	0.128
NLR, ratio	3.18 (1.88–5.12)	2.94 (1.73–4.40)	4.10 (2.34–7.20)	0.019
Albumin, g/L	39.05 ± 4.41	39.92 ± 3.81	37.56 ± 4.75	0.002
Total bilirubin, μmol/L	10.23 (8.00–13.80)	9.90 (7.79–13.05)	11.00 (8.20–14.98)	0.129
HCY, μmol/L	10.80 (8.70–14.40)	10.60 (8.65–14.15)	10.80 (8.50–14.40)	0.614
TG, mmol/	1.07 (0.76–1.48)	0.99 (0.76–1.41)	1.22 (0.76–1.63)	0.249
TC, mmol/L	4.62 (3.91–5.18)	4.76 (3.69–5.40)	4.53 (3.92–5.07)	0.408
HDL-C, mmol/L	1.27 (1.06–1.60)	1.32 (1.09–1.64)	1.16 (0.98–1.57)	0.037
LDL-C, mmol/L	2.80 (2.25–3.19)	2.80 (2.14–3.20)	2.80 (2.28–3.12)	0.837
ApoA, mmol/L	1.08 (0.94–1.21)	1.10 (0.98–1.23)	1.02 (0.88–1.19)	0.049
ApoB, mmol/L	0.93 (0.74–1.10)	0.93 (0.72–1.05)	0.93 (0.80–1.12)	0.199
ApoA/apoB, ratio	1.19 (0.97–1.46)	1.22 (1.00–1.60)	1.13 (0.90–1.37)	0.028

*Values are presented as numbers (%), means ± SD, or medians (interquartile range), p < 0.05 was considered statistically significant*.

*BMI, body mass index; mRS, modified Rankin scale; IQR, Interquartile range; NMDAR, N-methyl-D-aspartate receptor; LGI1, leucine-rich glioma inactivated protein 1; GABAB, gamma-aminobutyric acid B; CASPR2, contactin-associated protein-like 2; MOG, myelin oligodendrocyte glycoprotein; EEG, electroencephalogram; CRP, C-reactive protein; NLR, neutrophil-to-lymphocyte ratio; HCY, Homocysteine; TG, triglycerides; TC, total cholesterol; HDL-C high-density lipoprotein cholesterol; LDL-C, low-density lipoprotein cholesterol; apoA, apolipoprotein A; apoB, apolipoprotein B*.

Clinically, the median time from symptom onset until the treatment of patients with AE was 26 days (IQR: 13–63 days). Prodromal symptoms were found in 48 (35.6%) patients. Clinical symptoms of patients consisted of cognitive impairment (90.4%), mental behavior disorder (57.0%), epileptic seizures (76.3%), disturbance of consciousness (70.4%), central hypoventilation (11.1%), and autonomic nervous dysfunction (35.6%). Most patients developed 3 (IQR: 2–4) of these 6 categories of symptoms. Neuronal autoantibodies were examined in all patients. Anti-NMDAR antibodies were most common (42.2%), followed by anti-LGI-1 (31.9%), anti-GABABR (14.8%), anti-CASPR2 (5.9%), and anti-MOG antibodies (3.7%). Among the 135 patients with AE, 47 (34.8%) patients had weakly positive antibody titers, 63 (46.7%) patients had positive antibody titers, and 25 (18.5%) patients had strongly positive antibody titers. EEG examinations were abnormal in 94 (69.6%) patients and 22 (16.3%) patients had an underlying neoplasm.

### Factors Associated With Non-Response to Short-Term First-Line Treatment in AE Patients

Univariate analysis showed that higher mRS score at study entry and maximum mRS score (*p*= 0.032 and *p* < 0.001), higher proportion of anti-NMDAR subtypes (*p* = 0.043), lower proportion of LGI-1 subtypes (*p* = 0.004), strongly positive antibody titers (*p* = 0.004), higher NLR levels (*p* = 0.019), lower albumin levels (*p* = 0.002), lower HDL-C levels (*p* = 0.037), lower apoA levels (*p* = 0.049), and lower apoA/apoB levels (*p* = 0.049) were all associated with the non-response to short-term first-line treatment. No other differences were statistically significant (Table 1).

A multiple logistic regression model was used to analyze the factors associated with non-response to short-term first-line treatment (Table 2). Factors with a *p* < 0.06 in Table 1 were included in the final model. The multivariate logistic regression analysis showed that number of clinical symptoms [odds ratio (OR) 0.25, 95% CI 0.09–0.68, and *p* = 0.007], mental behavior disorder (OR 0.07, 95% CI 0.02–0.36, and *p* = 0.001), central hypoventilation (OR 0.04, 95% CI 0.00–0.56, and *p* = 0.017), maximum mRS score (OR 9.12, 95% CI 2.40–34.66, and *p* = 0.001), and albumin level (OR 0.85, 95% CI 0.75–0.96, and *p* = 0.010) were significantly associated with non-response to short-term first-line treatment. The other risk factors did not retain significance in the final model (Table 2).

**Table 2:** Multivariate analysis of factors associated with poor short-term first-line treatment response.

**Variable**	**OR (95% CI)**	***P*** **-value**
Median time from symptom onset until	1.00 (0.99–1.00)	0.683
treatment, days		
Number of clinical symptoms	0.24 (0.08–0.67)	0.006
Mental behavior disorder	0.07 (0.01–0.34)	0.001
Disturbance of consciousness	0.23 (0.04–1.32)	0.100
Central hypoventilation	0.04 (0.00–0.52)	0.015
Autonomic nervous dysfunction	0.21 (0.05–1.01)	0.052
MRS score at study entry	0.54 (0.18–1.65)	0.281
Maximum mRS score	8.40 (2.18–32.28)	0.002
NMDAR-Abs	0.49 (0.08–3.03)	0.446
LGI-1-Abs	1.00 (0.15–6.81)	0.998
Antibody titers	0.66 (0.21–2.05)	0.471
Strongly positive	0.42 (0.05–3.98)	0.453
NLR	1.01 (0.91–1.12)	0.841
Albumin	0.85 (0.75–0.97)	0.013
HDL-C	3.21 (0.26–39.11)	0.361
ApoA	0.52 (0.01–30.15)	0.753

*mRS, modified Rankin scale; NMDAR, N-methyl-D-aspartate receptor; LGI-1, leucine-rich glioma inactivated protein 1; NLR, neutrophil-to-lymphocyte ratio; HDL-C high-density lipoprotein cholesterol; apoA, apolipoprotein A*.

### Subgroup Analysis of Factors Associated With Non-Response to Short-Term First-Line Treatment in Anti-NMDAR Encephalitis

To further analyze the factors influencing short-term first-line treatment response of subtypes of AE, we performed subgroup analysis based on antibody types. Due to the small number of cases, we performed factor analysis only in anti-NMDAR subtypes, which with a relatively large number of cases. There were 55 anti-NMDAR encephalitis patients in our study, 24 (43.6%) patients were responders and 31 (56.4%) were non-responders. Univariate analysis showed that more clinical symptoms (*p* < 0.001), mental behavior disorder (*p* = 0.002), central hypoventilation (*p* = 0.001), autonomic nervous dysfunction (*p* = 0.001), higher mRS score at study entry and maximum mRS score (*p* = 0.001 and *p* < 0.001), strongly positive antibody titers (*p* = 0.035), and higher NLR levels (*p* = 0.026) were all associated with non-response to short-term first-line treatment. No other differences were statistically significant (Supplementary Table 1).

All factors with a *p* < 0.06 in Supplementary Table 1 were included in the multivariate logistic regression model. The multivariate logistic regression analysis showed that mental behavior disorder (OR 0.04, 95% CI 0.00–0.72, and *p* = 0.029), autonomic nervous dysfunction (OR 0.09, 95% CI 0.01–0.94, and *p* = 0.044), and maximum mRS score (OR 23.37, 95% CI 1.54–354.85, and *p* = 0.023) were still associated with non-response to short-term first-line treatment in patients with anti-NMDAR encephalitis. The other risk factors did not retain significance in the final model (Supplementary Table 2).

### Treatments and Prognosis of AE Patients

Among the 135 patients with AE, 128 (94.8%) patients were received first-line immunotherapy, 17 (12.6%) patients received second-line immunotherapy, and 7 (5.2%) patients did not receive any immunotherapy (Figure 1 and Table 3). The median length of hospital stay was 15 (IQR: 11–21) days. First-line immunotherapy in this study included intravenous glucocorticoid therapy and IVIG treatment. In total, 53 (39.3%) patients received glucocorticoids, 10 (7.4%) patients received IVIG, and 65 (48.1%) patients received a combination of glucocorticoid and IVIG treatment. The proportion of patients who received a combination of glucocorticoid and IVIG in the non-response group (61%) was higher than that in the response group (42%).

**Table 3:** Treatment and outcomes of patients with AE.

**Variable**	**All** **(*****n*** **= 135)**	**Responses group** **(*****n*** **= 69)**	**Non-response group** **(*****n*** **= 59)**	***P*** **-value**
Length of hospital stay, days	15 (11–21)	14 (12–19)	16 (12–25)	0.064
First-line immunotherapy, n (%)	128 (94.8)	-	-	-
Steroids	53 (39.3)	36 (52.2)	17 (28.8)	0.007
IVIG	10 (7.4)	4 (5.8)	6 (10.2)	0.556
Combined	65 (48.1)	29 (42)	36 (61)	0.032
Second-line immunotherapy, *n* (%)	17 (12.6)	2 (2.9)	15 (25.4)	<0.001
Relapses^a^, n (%, total *n* = 121)	18 (13.3)	5 (8.9)	12 (24.5)	0.031
Relapses^b^, n (%, total *n* = 80)	19 (14.1)	5 (13.2)	13 (31.7)	0.050

*Values are presented as numbers (%), or medians (interquartile range), p < 0.05 was considered statistically significant*.

*IQR, Interquartile range; IVIG, intravenous immunoglobulins*.

**Relapses^a^:** *: clinical relapses in 12 months*.

**Relapses^b^:** *: clinical relapses in 24 months*.

**Figure 2:**
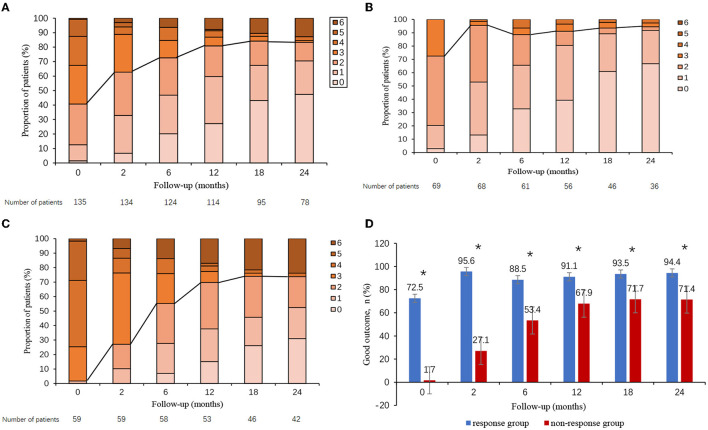
Clinical outcome in autoimmune encephalitis patients. Clinical outcome in all patients **(A)**, patients who responded to short-term first-line immunotherapy **(B)**, patients who failed short-term first-line immunotherapy **(C)**, and overall proportion of patients with good outcomes according to short-term first-line treatment response **(D)**. The proportion of patients with good outcomes was significantly lower in non-responders than in responders. *p < 0.05.

All 135 patients were followed up, of whom 78 patients had been followed-up for 24 months (Figure 2A). We evaluated the patient's outcome with the mRS, and the whole follow-up process is shown in Figure 2A. In general, most patients achieved a good outcome (mRS ≤ 2), and the proportion of patients with good outcomes increased with longer follow-up. This trend was noted in Figure 2A showing that the proportion of patients with good outcomes was 40.7% (55/135) at discharge, 63.4% (85/134) at 2 months, 71.8% (89/124) at 6 months, 79.8% (91/114) at 12 months, 82.1% (78/95) at 18 months, and 82.1% (64/78) at 24 months. During the 12-month follow-up, 18 (13.3%) of 121 patients had a clinical relapse. During the 24-month follow-up, 19 (14.1%) of 80 patients had a clinical relapse (Table 3).

Outcomes were compared between responders and non-responders. In general, non-responders overall did worse long-term than responders. The whole mRS distribution of patients in follow-up could be found in Figure 2B (responders) and Figure 2C (non-responders). Figure 2D presented the proportion of patients with good outcomes for responders and non-responders in follow-up. As compared with responders, non-responders had a significantly lower proportion of good outcomes during all times of followed-up (*p* < 0.05, all). Patients in the non-response group had a higher frequency of relapses than did those in the response group during both the 12- and 24-month follow-ups (24.5 vs. 8.9%, *p* = 0.031; 31.7 vs. 13.2%, *p* = 0.050) (Table 3).

To further evaluate whether short-term first-line treatment response can predict the long-term outcomes, we put the unbalanced characteristics together with first-line treatment response into a univariate logistic regression model (Table 4). Multivariate logistic regression analysis showed that increasing age and poor first-line treatment response were significantly associated with the poor long-term prognosis of patients with AE, both at 12-month and 24-month follow-ups (Table 5).

**Table 4:** Univariate analysis of factors associated with poor prognosis in patients with AE.

**Variable**	**12-Month prognosis**		**24-Month prognosis**	
	**OR (95% CI)**	***P*** **-value**	**OR (95% CI)**	***P*** **-value**
Age	0.96 (0.94–0.99)	0.021	0.95 (0.91–0.99)	0.014
Sex, female,	0.71 (0.27–1.83)	0.476	0.96 (0.30–3.10)	0.951
MRS score at study entry	0.44 (0.20–0.95)	0.036	0.89 (0.38–2.06)	0.786
NMDAR-Abs	0.67 (0.26–1.76)	0.419	0.43 (0.13–1.43)	0.170
LGI-1-Abs	0.29 (0.08–1.04)	0.057	0.20 (0.02–1.61)	0.130
Albumin	1.00 (0.90–1.11)	1.000	1.09 (0.96–1.23)	0.179
First-line immunotherapy				
Steroids	0.98 (0.37–2.60)	0.971	0.87 (0.26–2.89)	0.816
IVIG	1.81 (0.43–7.63)	0.422	2.64 (0.57–12.16)	0.214
Combined	0.81 (0.32–2.07)	0.664	0.71 (0.22–2.26)	0.556
Second-line immunotherapy	0.90 (0.23–3.48)	0.877	0.72 (0.14–3.66)	0.694
First-line treatment response	4.82 (1.63–14.25)	0.004	6.80 (1.41–32.86)	0.017

*mRS, modified Rankin scale; NMDAR, N-methyl-D-aspartate receptor; LGI-1, leucine-rich glioma inactivated protein 1; IVIG, intravenous immunoglobulins*.

**Table 5:** Multivariate analysis of factors associated with poor prognosis in patients with AE.

**Variable**	**12-Month prognosis**		**24-Month prognosis**	
	**OR (95% CI)**	***P*** **-value**	**OR (95% CI)**	***P*** **-value**
Age	0.96 (0.93–0.99)	0.016	0.95 (0.91–0.99)	0.020
MRS score at study entry	0.46 (0.20–1.05)	0.066	0.91 (0.37–2.24)	0.842
First-line immunotherapy	1.12 (0.63–1.99)	0.693	1.20 (0.58–2.46)	0.626
Second-line immunotherapy	0.84 (0.17–4.06)	0.825	0.81 (0.12–5.28)	0.823
First-line treatment response	4.74 (1.44–15.59)	0.010	8.81 (1.65–47.16)	0.011

*mRS, modified Rankin scale*.

Furthermore, we performed the prognostic analysis in patients with anti-NMDAR subtypes. At discharge and at 2-, 6-, and 12-month follow-ups, the proportion of patients with good outcomes in the non-response group was all significantly lower than that in the response group (*p* < 0.001, *p* < 0.001, *p* < 0.001, and *p* = 0.001, respectively). At 18- and 24-month follow-ups, there was no significant difference in the outcomes between the response group and the non-response group (*p* = 0.069 and *p* = 0.139). There was no significant difference in the frequency of relapses between the two groups during both the 12- and 24-month follow-ups (*p* = 0.063 and *p* = 0.067) (Supplementary Table 3).

### Treatment and Prognosis of AE Patients Who Failed Short-Term First-Line Immunotherapy

Of the 59 non-responders, 32 (54.2%) had no further immunotherapy, 12 (20.3%) had a repetition of first-line immunotherapy, and 15 (25.4%) received second-line immunotherapy (Figure 1). To compare the effects of the further treatment, we compared the basic characteristics between the three groups as shown in Table 6. Patients who received second-line treatment were younger (*p* = 0.007). No difference was found in sex, BMI, the median time from symptom onset until treatment, MRS score at study entry, maximum mRS score, mRS score after short-term first-line treatment, and antibody titers among the three groups.

**Table 6:** Characteristics and outcomes of patients with AE who failed short-term first-line immunotherapy.

**Variable**	**No additional treatment group** **(*****n*** **= 32)**	**Repetition of first-line treatment group (*n* = 12)**	**Second-line treatment group** **(*****n*** **= 15)**	***P*** **-value**
Age (year), median (IQR)	51 (35–65)	57 (35–67)	26 (15–49)	0.007
Sex, female, *n* (%)	16 (50)	3 (25)	9 (60)	0.178
BMI (Kg/m^2^), median (IQR)	23.49 ± 2.75	23.95 ± 2.25	21.67 ± 2.75	0.052
Median time from symptom onset until treatment, days (IQR)	23 (12–35)	20 (6–40)	23 (10–32)	0.476
MRS at study entry, median (IQR)	4 (3–4)	4.5 (4–5)	4 (4–5)	0.151
Maximum mRS, median (IQR)	4.5 (4–5)	5 (4–5)	5 (4–5)	0.781
MRS after short-term first-line treatment, median (IQR)	4 (4–5)	4 (4–5)	5 (4–5)	0.325
Antibody titers, *n*, (%)				
Weakly positive	11 (34.4)	4 (33.3)	1 (6.7)	0.103
Positive	14 (43.8)	2 (16.7)	9 (60.0)	0.078
Strongly positive	7 (21.9)	6 (50)	5 (33.3)	0.189
Relapses^a^, n (%, total *n* = 48)	9 (36)	1 (11.1)	2 (14.3)	0.264
Relapses^b^, n (%, total *n* = 38)	9 (42.9)	1 (12.5)	3 (25.0)	0.242

*Values are presented as numbers (%), means ± SD, or medians (interquartile range), p < 0.05 was considered statistically significant*.

*BMI, body mass index; mRS, modified Rankin scale; IQR, Interquartile range*.

**relapses^a^:** *: clinical relapses in 12 months*.

**relapses^b^:** *: clinical relapses in 24 months*.

**Figure 3:**
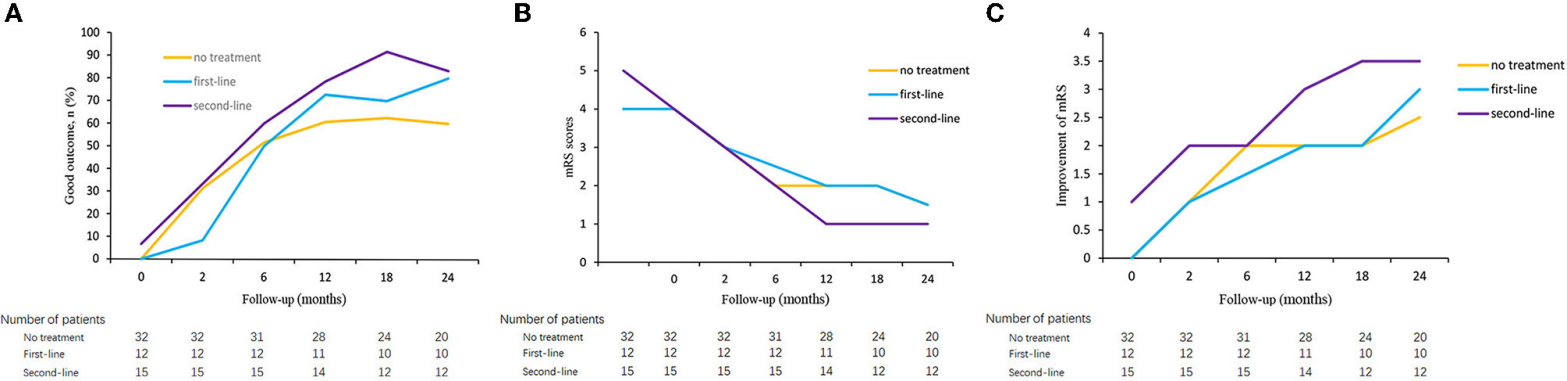
Clinical outcome in autoimmune encephalitis patients who failed short-term first-line treatment. Good outcome of patients who failed first-line treatment in three groups **(A)**, mRS score of patients who failed first-line treatment in three groups **(B)**, and improvement of mRS score of patients who failed first-line treatment in three groups **(C)**.

As shown in Figure 3, the overall trend of the three groups' non-responders was a gradual increase in the proportion of good outcomes and a decrease in the mRS score. The improvement of mRS scores increased with the increase of follow-up time. Outcomes were compared between the three groups. The proportion of good outcomes in non-responders with second-line treatment was higher than other non-responders. The median mRS score of non-responders with second-line treatment decreased more rapidly than other non-responders. The median mRS score was lower in patients with second-line treatment group at 12-, 18- and 24-month follow-ups. However, the rate of a good outcome and median mRS score was not significantly different among the three groups (*p* > 0.05). Patients with second-line treatment had a higher improvement of mRS score than other non-responders, and the difference was significant at discharge, at 12-month and 18-month follow-ups (*p* = 0.011, *p* = 0.008, and *p* = 0.041, respectively). There was no difference in the rate of relapses among the three groups, both at 12-month and 24-month follow-ups (Table 6).

To further evaluate the efficacy of the second-line treatment, we compared the proportion of good outcomes in non-responders with second-line treatment and the responders. We found that non-responders with second-line treatment had poor outcomes than responders within 6 months (*p* > 0.05) but no significant difference was found between the two groups after 6 months during long-term follow-up (*p* > 0.05, all) (Supplementary Table 4).

## Discussion

Autoimmune encephalitis (AE) is a potentially life-threatening neurologic disease and can be reversed with early active treatment (2, 4). The findings of our study provided several relevant insights regarding the characteristics of patients who failed short-term (10–14 days) first-line immunotherapy, and their long-term prognosis. These insights may increase awareness of the potential factors that may predict and affect the risk of poor initial treatment response in patients with AE, providing a basis for early adjustment of treatment.

The hypothesis of whether clinical data may provide early clues in predicting the initial treatment response in patients with AE is fascinating. Mental behavior disorder is one of the main manifestations of patients with AE (4, 18, 20), which has been reported to be associated with disease severity (20). We also found that mental behavior disorder was associated with non-response to short-term first-line treatment in this study. In addition to mental behavior disorder, we also found central hypoventilation and autonomic nervous dysfunction were also associated with poor treatment response in patients with AE (2). There has been emerging evidence in the literature suggesting central hypoventilation and autonomic dysfunction were associated with disease severity and poor outcome (5, 10, 11, 20, 21). Our team also had found these symptoms were indicative of a failure to respond to first-line therapy in anti-NMDAR encephalitis (22). Combined with this study, we hypothesize that they can also be indicators for limited initial treatment response in patients with AE. Other than these symptoms, epileptic seizure and cognitive impairment were also common in patients with AE, and in our study. But we did not find an association between these characters and short-term first-line treatment response. In our study, we also found that the increasing age was significantly associated with a poorer long-term prognosis of patients with AE. This conclusion is in line with several previous studies (23–25). However, no association was found between age and short-term first-line treatment response in this study.

To determine whether AE subtypes affect the treatment response, we compared the proportion of each antibody between the response group and the non-response group. We found anti-NMDAR encephalitis patients tended to have a poor first-line treatment response, while anti-LGI-1 encephalitis patients tend to have a good response. This is in accordance with a previous study that focused on disease outcome, which found that anti-LGI-1 encephalitis patients responded better to treatment and had a relatively better prognosis than anti-NMDAR encephalitis patients (26). In addition, there was a trend toward a higher antibody titer in the non-response group in patients with AE, and in anti-NMDAR encephalitis patients. However, antibody titer was not an independent predictor of poor short-term first-line treatment response by multivariate analysis. In our previous study, we also did not find an association between the antibody titers and treatment response in anti-NMDAR encephalitis patients (22). At present, studies on the relationship between antibody titer and patients with AE are inconsistent. Some studies have found an association between antibody titer and severity or outcome of patients with AE (11, 27, 28), while others have found no association (2, 10, 29). Further studies, especially large prospective studies, are needed to better understand the association between antibody titer and this disease.

As a biomarker of systemic inflammation, NLR has been defined as a biomarker for assessing disease activity and prognosis in patients with autoimmune diseases, such as neuromyelitis optica spectrum disorder and multiple sclerosis (MS) (30–32). Recently, studies found that NLR may be a biomarker to monitor disease progression in patients with AE (3, 33). Our team also found that it is associated with the first-line treatment response in anti-NMDAR encephalitis (22). In this study, we found that higher NLR level was associated with non-response to short-term first-line treatment in patients with AE, and in anti-NMDAR subtype encephalitis patients. But it was not an independent influence factor of initial treatment response in multivariate analysis. We guess NLR may be a potential biomarker for assessing early treatment response in patients with AE. In our study, the lower albumin level was an independent predictor of poor short-term first-line treatment in patients with AE, which is in agreement with the previous study by our team in a patient with anti-NMDAR encephalitis (22). Jang et al. (34) also found that high albumin level is a predictor of favorable response to immunotherapy in patients with AE. But, Mo et al. (23) found no association between albumin and poor prognosis in patients with anti-NMDAR encephalitis. Further studies about the association between albumin levels and AE are needed. Growing evidence suggests an association between dyslipidemia and many autoimmune diseases (35–37). Previous studies have found that lipid profiles were associated with the severity and prognosis of patients with anti-NMDAR encephalitis (38, 39). In this study, low HDL-C, apoA, and apoA/apoB levels were also associated with non-response to short-term first-line treatment. But they were not independent predictors of poor short-term first-line treatment response in patients with AE. Therefore, further studies about the association between lipid profiles and AE are needed.

Decisions about the type and duration of immunotherapy were made by physicians and were based on clinical symptoms. Patients who were severer and younger tended to have more aggressive treatment in our study. To assess patients' outcomes, all patients were followed for a long period of time, not just at discharge. In this study, we found non-responders overall did worse long-term than responders, and first-line treatment responses were significantly correlated with the long-term prognosis of patients with AE. Similar results were obtained in the previous study, which found patients who improved with the first-line treatment for 4 weeks usually had a better outcome than those who did not (4). Non-response to short-term first-line treatment can be used as an indicator of poor long-term prognosis in patients with AE. This provides a basis for clinicians to assess patients' prognoses early.

Further treatment of patients with non-response to short-term first-line treatment is a concern and challenge for clinicians. In this study, we found non-responders with second-line treatment had a higher improvement of mRS score than other non-responders, but there was no significant difference in the rate of good outcomes or the median mRS score. This finding might be associated with more active immunotherapy in the severe patients in this study, leading to an illusion that the more aggressive the treatment, the worse the outcome. That's because a patient's condition directly affects doctors' decisions about additional treatment. Even though all these patients remained in a similar neurological status (mRS score) after failing first-line immunotherapy, selection bias, subtypes of AE, and other confounding factors cannot be ruled out in this retrospective study. Previous studies have found the positive therapeutic effect of second-line immunotherapy in patients with AE (4, 13, 16). Combined with our findings that patients who further received second-line treatment had a higher improvement of mRS scores than other non-responders. We speculate that second-line therapy is a more aggressive treatment option for patients who failed with short-term first-line therapy.

To our knowledge, a systematic analysis of the characteristics and prognosis of patients with AE who failed short-term first-line immunotherapy has not been previously done. In this study, we did a systematic analysis in patients with AE and also in anti-NMDAR subtypes. The identification of clinical factors in the early stage of the disease that is potentially associated with poor initial treatment response may help clinicians to assess the prognosis of patients and guide the use of a more targeted aggressive immunotherapy early. In addition, we also conducted a preliminary discussion and analysis on the further treatment options and prognosis of patients who failed short-term first-line treatment, which is also a concern and challenge for clinicians. Although our study has the limitation of non-randomization in treatment options and many confounders, it provides a basis for future randomized controlled trials.

Our study has several potential limitations. First, this study is an uncontrolled retrospective analysis, many confounders, especially the treatment options, selection bias cannot be ruled out. On the other hand, due to our limited number of cases, further analysis or multiple testing of potential effective factors is not possible. Another drawback of our study was the merging of all AE subtypes together, we just performed factor analysis in anti-NMDAR subtypes, which with a relatively large number of cases. For other subtypes, the stratified analysis could not be performed, further study is needed. In addition, the outcomes of patients in our study were assessed just by mRS score, which may not be precise and are probably not ideal for detecting non-motor sequelae, such as neuropsychological sequelae.

In conclusion, our study found that disease severity, clinical features, anti-NMDAR subtypes, antibody titers, NLR, albumin, HDL-C, and apoA levels were all associated with non-response to short-term first-line treatment. Patients who had non-response to the short-term first-line immunotherapy tended to have a poor long-term outcome. Second-line immunotherapy may be an aggressive treatment option for patients who failed short-term first-line immunotherapy. Our findings may help clinicians to assess patients' prognoses in the early stages of the disease and guide the use of a more targeted aggressive immunotherapy. To further improve and validate our findings, prospective randomized controlled studies are necessary.

## Data Availability Statement

The raw data supporting the conclusions of this article will be made available by the authors, without undue reservation.

## Ethics Statement

The studies involving human participants were reviewed and approved by Ethics Committee of the Shandong Provincial Hospital Affiliated to Shandong University. Written informed consent from the participants' legal guardian/next of kin was not required to participate in this study in accordance with the national legislation and the institutional requirements.

## Author Contributions

FL, CW, and SG contributed to the conception and design of the study. FL, TH, and BW collected the data. BZ performed the statistical analysis. FL wrote the first draft of the manuscript. MH and SG critically revised the manuscript. All authors have read and approved the final manuscript.

## Funding

This study was supported by the National Natural Science Foundation of China (No. 82072079).

## Conflict of Interest

The authors declare that the research was conducted in the absence of any commercial or financial relationships that could be construed as a potential conflict of interest.

## Publisher's Note

All claims expressed in this article are solely those of the authors and do not necessarily represent those of their affiliated organizations, or those of the publisher, the editors and the reviewers. Any product that may be evaluated in this article, or claim that may be made by its manufacturer, is not guaranteed or endorsed by the publisher.
