# Supplementary material for: Influential Factors, Treatment and Prognosis of Autoimmune Encephalitis Patients With Poor Response to Short-Term First-Line Treatment
Source: Front Neurol. 2022 Apr 14;13:861988. doi: 10.3389/fneur.2022.861988 (PMC9046540; doi:10.3389/fneur.2022.861988)
Supplement: Supplementary file 1 [file Table_1.DOCX]

**Supplementary Table 1** Univariate analysis of factors associated with poor short-term first-line treatment response in anti-NMDAR encephalitis

| Variable | Responses  (n=24) | Non-response  (n=31) | P1-value |
| --- | --- | --- | --- |
| Age, years | 30 (25-35) | 33 (19-45) | 0.628 |
| Sex, female, n (%) | 16 (66.7) | 16 (51.6) | 0.262 |
| BMI, Kg/m^2^ | 21.78(19.95-23.48) | 22.58(19.74-24.69) | 0.703 |
| Median time from symptom onset until treatment, days | 15(8-32) | 20(12-31) | 0.486 |
| Prodromal symptoms at presentation, n (%) | 12 (50) | 17(54.8) | 0.721 |
| Number of clinical symptoms | 3 (2-4) | 5 (3-5) | ＜0.001 |
| Clinical symptoms, n (%) |  |  |  |
| mental behavior disorder | 13 (54.2) | 28 (90.3) | 0.002 |
| epileptic seizure | 19 (79.2) | 23 (74.2) | 0.667 |
| disturbance of consciousness | 15 (62.5) | 26 (83.9) | 0.071 |
| cognitive impairment | 21 (87.5) | 31 (100) | 0.077 |
| central hypoventilation | 0 (0) | 11 (35.5) | 0.001 |
| autonomic nervous dysfunction | 5 (20.8) | 21 (67.7) | 0.001 |
| MRS at study entry, scores | 4 (3-4) | 4 (4-5) | 0.001 |
| Maximum mRS, scores | 4 (4-4) | 5 (4-5) | ＜0.001 |
| Antibody titers, n, (%) |  |  | 0.073 |
| weakly positive | 10 (41.7) | 6 (19.4) | 0.071 |
| positive | 9 (37.5) | 10 (32.3) | 0.685 |
| strongly positive | 5 (20.8) | 15 (48.4) | 0.035 |
| Abnormal EEG, n (%) | 14 (58.3) | 21 (67.7) | 0.472 |
| Tumor comorbidity, n (%) | 3 (12.5) | 7 (22.6) | 0.486 |
| CRP, mg/L | 2.74(0.86-7.31) | 5.00(1.0-11.52) | 0.492 |
| NLR, ratio | 2.43(1.71-3.18) | 3.71(2.09-7.03) | 0.026 |
| Albumin, g/L | 40.75(37.2843.78) | 38.7(35.4-42.9) | 0.373 |
| Total bilirubin, μmol/L | 8.98(7.63-11.86) | 11.8(7.10-16.3) | 0.076 |
| HCY, μmol/L | 10.6(7.38-14.43) | 11.9(8.5-15.3) | 0.476 |
| TG, mmol/ | 1.33±0.60 | 1.23±0.56 | 0.537 |
| TC, mmol/L | 4.66(3.81-5.02) | 4.46(3.88-4.75) | 0.425 |
| HDL-C, mmol/L | 1.12(1.00-1.37) | 1.13(0.98-1.26) | 0.430 |
| LDL-C, mmol/L | 2.85(2.18-3.20) | 2.58(2.28-3.00) | 0.570 |
| ApoA, mmol/L | 1.04±0.23 | 0.97±0.19 | 0.192 |
| ApoB, mmol/L | 0.96(0.71-1.04) | 0.93(0.74-1.13) | 0.420 |
| ApoA/apoB, ratio | 1.11(0.93-1.55) | 1.09(0.90-1.31) | 0.281 |

Values are presented as numbers (%), means ± SD, or medians (interquartile range), p<0.05 was considered statistically significant

Abbreviations: BMI: body mass index; mRS: modified Rankin scale; IQR: Interquartile range; EEG: electroencephalogram; CRP, C-reactive protein; NLR, neutrophil-to-lymphocyte ratio; HCY, Homocysteine; TG, triglycerides; TC, total cholesterol; HDL-C high-density lipoprotein cholesterol; LDL-C, low-density lipoprotein cholesterol; apoA, apolipoprotein A; apoB, apolipoprotein B.
