# Supplementary material for: Influential Factors, Treatment and Prognosis of Autoimmune Encephalitis Patients With Poor Response to Short-Term First-Line Treatment
Source: Front Neurol. 2022 Apr 14;13:861988. doi: 10.3389/fneur.2022.861988 (PMC9046540; doi:10.3389/fneur.2022.861988)
Supplement: Supplementary file 2 [file Table_2.DOCX]

**Supplementary Table 2.** Multivariate analysis of factors associated with poor short-term first-line treatment response in anti-NMDAR encephalitis

| Variable | OR (95% CI) | P-value |
| --- | --- | --- |
| Number of clinical symptoms | 0.49 (0.13-1.92) | 0.308 |
| Mental behavior disorder | 0.04 (0.00-0.72) | 0.029 |
| Autonomic nervous dysfunction | 0.09 (0.01-0.94) | 0.044 |
| MRS score at study entry | 0.49 (0.07-3.58) | 0.478 |
| Maximum mRS score | 23.37 (1.54-354.85) | 0.023 |
| Strongly positive antibody titers | 0.50 (0.06-3.95) | 0.513 |
| NLR | 0.87 (0.66-1.15) | 0.328 |

Abbreviations: mRS: modified Rankin scale; NLR, neutrophil-to-lymphocyte ratio.
