# Supplementary material for: Influential Factors, Treatment and Prognosis of Autoimmune Encephalitis Patients With Poor Response to Short-Term First-Line Treatment
Source: Front Neurol. 2022 Apr 14;13:861988. doi: 10.3389/fneur.2022.861988 (PMC9046540; doi:10.3389/fneur.2022.861988)
Supplement: Supplementary file 3 [file Table_3.DOCX]

**Supplementary Table3.** Treatment and outcomes of anti-NMDAR encephalitis

| Variable | Responses group  (n=24) | Non-response group  (n=31) | P-value |
| --- | --- | --- | --- |
| Length of hospital stay, days | 18 (14-22) | 22 (15-39) | 0.063 |
| First-line immunotherapy, n (%) |  |  |  |
| steroids | 9 (37.5) | 9 (29.0) | 0.507 |
| IVIG | 3 (12.5) | 4 (12.9) | 1.000 |
| combined | 12 (50.0) | 18 (58.1) | 0.551 |
| Second-line immunotherapy, n (%) | 1 (4.2) | 11 (35.5) | 0.005 |
| Good outcome, n (%) |  |  |  |
| at discharge | 16 (66.7) | 0 (0.0) | ＜0.001 |
| at 2 months | 24 (100) | 10 (32.3) | ＜0.001 |
| at 6 months | 22 (100) | 17 (54.8) | ＜0.001 |
| at 12 months | 19 (100) | 21 (72.4) | 0.015 |
| at 18 months | 17 (100) | 22 (78.6) | 0.069 |
| at 24 months | 15 (100) | 21 (80.8) | 0.139 |
| Relapses^1^, n (%, total n=45) | 0 (0.0) | 5 (19.2) | 0.063 |
| Relapses^2^, n (%, total n=38) | 0 (0.0) | 4 (17.4) | 0.138 |

Values are presented as numbers (%), or medians (interquartile range), p<0.05 was considered statistically significant.

Abbreviations: IVIG: intravenous immunoglobulins;

relapses^1^: clinical relapses in 12 months;

relapses^2^: clinical relapses in 24 months.
