# Supplementary material for: Influential Factors, Treatment and Prognosis of Autoimmune Encephalitis Patients With Poor Response to Short-Term First-Line Treatment
Source: Front Neurol. 2022 Apr 14;13:861988. doi: 10.3389/fneur.2022.861988 (PMC9046540; doi:10.3389/fneur.2022.861988)
Supplement: Supplementary file 4 [file Table_4.DOCX]

**Supplementary Table 4.** Outcomes of responders and non-responders with second-line treatment

| Variable | Responses group  (n=69) | Non-responders with Second-line group  (n=15) | P-value |
| --- | --- | --- | --- |
| Good outcome, n (%) |  |  |  |
| at discharge | 50 (72.5) | 1 (6.7) | ＜0.001 |
| at 2 months | 65 (95.6) | 5 (33.3) | ＜0.001 |
| at 6 months | 54 (88.5) | 9 (60.0) | 0.017 |
| at 12 months | 51(91.1) | 11 (78.6) | 0.193 |
| at 18 months | 43 (93.5) | 11 (91.7) | 1.000 |
| at 24 months | 34 (94.4) | 10 (83.3) | 0.257 |
| Relapses^1^, n (%, total n=121) | 5 (8.9) | 2 (14.3) | 0.621 |

Values are presented as numbers (%), p<0.05 was considered statistically significant.

relapses^1^: clinical relapses in 12 months;
